# Antagonizing CD105 enhances radiation sensitivity in prostate cancer

**DOI:** 10.1038/s41388-018-0278-0

**Published:** 2018-05-02

**Authors:** Anisha Madhav, Allen Andres, Frank Duong, Rajeev Mishra, Subhash Haldar, Zhenqiu Liu, Bryan Angara, Roberta Gottlieb, Zachary S. Zumsteg, Neil A. Bhowmick

**Affiliations:** 1 Department of Biomedical Sciences, Cedars-Sinai Medical Center, Los Angeles, CA 90048 USA; 2 Department of Medicine, Cedars-Sinai Medical Center, Los Angeles, CA 90048 USA; 3 Department of Radiation Oncology, Cedars-Sinai Medical Center, Los Angeles, CA 90048 USA; 4 Greater Los Angeles Veterans Administration, Los Angeles, CA 90048 USA

## Abstract

Radiation therapy is the primary intervention for nearly half of the patients with localized advanced prostate cancer and standard of care for recurrent disease following surgery. The development of radiation-resistant disease is an obstacle for nearly 30–50% of patients undergoing radiotherapy. A better understanding of mechanisms that lead to radiation resistance could aid in the development of sensitizing agents to improve outcome. Here we identified a radiation-resistance pathway mediated by CD105, downstream of BMP and TGF-β signaling. Antagonizing CD105-dependent BMP signaling with a partially humanized monoclonal antibody, TRC105, resulted in a significant reduction in clonogenicity when combined with irradiation. In trying to better understand the mechanism for the radio-sensitization, we found that radiation-induced CD105/BMP signaling was sufficient and necessary for the upregulation of sirtuin 1 (SIRT1) in contributing to p53 stabilization and PGC-1α activation. Combining TRC105 with irradiation delayed DNA damage repair compared to irradiation alone. However, in the absence of p53 function, combining TRC105 and radiation resulted in no reduction in clonogenicity compared to radiation alone, despite similar reduction of DNA damage repair observed in p53-intact cells. This suggested DNA damage repair was not the sole determinant of CD105 radio-resistance. As cancer cells undergo an energy deficit following irradiation, due to the demands of DNA and organelle repair, we examined SIRT1’s role on p53 and PGC-1α with respect to glycolysis and mitochondrial biogenesis, respectively. Consequently, blocking the CD105-SIRT1 axis was found to deplete the ATP stores of irradiated cells and cause G2 cell cycle arrest. Xenograft models supported these findings that combining TRC105 with irradiation significantly reduces tumor size over irradiation alone (*p* value = 10^−9^). We identified a novel synthetic lethality strategy of combining radiation and CD105 targeting to address the DNA repair and metabolic addiction induced by irradiation in p53-functional prostate cancers.

## Introduction

Prostate cancer is the second leading cause of cancer mortality in men. The standard of care for localized prostate cancer is radiotherapy or surgical resection. Radiation is also used as an adjuvant therapy following surgery, salvage therapy after biochemical recurrence, and for palliation in the setting of distant metastasis. Up to 30% of localized prostate cancer patients treated with definitive radiation therapy develop recurrent radio-resistant disease and the most common anatomic site of recurrence is within the prostate itself, even in patients at high risk of metastasis [1–3]. Further, 50% of patients that undergo salvage radiation therapy after biochemical recurrence will have disease progression [4]. Although dose escalation improves biochemical control, toxicity remains a significant obstacle in optimizing local control [5, 6]. Accordingly, sensitizing agents are needed to improve tumor eradication and minimize toxicity to normal structures. With the rational that targeting mechanisms of radio-resistance can yield durable sensitization, we identified a novel pathway affecting both DNA repair and energy demands manifested by irradiation of prostate cancer cells.

Endoglin (CD105), a type III transforming growth factor-beta/bone morphogenic protein (TGF-β/BMP) co-receptor, recognized as a marker of proliferating endothelia, is upregulated in several cancers, including prostate cancer [7]. CD105 behaves like a switch on the cell surface to inhibit TGF-β signaling and promote BMP signaling. Therefore, silencing or knocking out CD105 results in the gain of TGF-β-mediated Smad2/3 signaling and loss of Smad1/5 signaling associated with BMP activity [8]. CD105 expression on various cancers has correlated with progression, metastasis, aggressiveness, and evasion to conventional therapeutics [9–12]. Various DNA repair genes were found to be downregulated by CD105 silencing, thereby sensitizing ovarian cancer to DNA a damaging agent, cisplatin [13]. However, these studies did not distinguish between the CD105 effects on TGF-β and BMP signaling on DNA damage repair. Significant data are reported for the use of specific TGF-β inhibition in radiation sensitizing breast cancer and glioblastoma [14, 15]. However, limited information is known about the role of BMP signaling in response to radiation. In this study, we use TRC105, a partially humanized monoclonal antibody that blocks the CD105/BMP signaling complex. Importantly, as TRC105 does not affect the CD105/TGF-β signaling axis, the role of CD105/BMP signaling on radiation responsiveness was tested [16]. Based on our finding that CD105 was elevated by irradiation, we hypothesized targeting CD105, using TRC105, could sensitize prostate cancer to irradiation. Of note, numerous phase I trials have shown TRC105 to be well tolerated, but it has had limited therapeutic efficacy for prostate cancer as a single agent [17, 18].

Probing CD105/BMP regulation of DNA repair genes led us to identify sirtuin 1 (SIRT1), a NAD^+^-dependent histone deacetylase, as a BMP-regulated target. SIRT1 activation is observed in prostate cancer and in response to irradiation [19]. In the context of cancer, SIRT1 has been studied primarily for its role in DNA damage response. Outside of cancer biology, SIRT1 de-regulation is associated with metabolic, neurodegenerative, and cardiovascular diseases [20–22]. SIRT1 has both tumor suppressor and oncogenic properties [23]. Apart from histones, SIRT1 regulates p53 and peroxisome proliferator-activated receptor gamma co-activator 1-alpha (PGC-1α) [22, 24, 25]. SIRT1-mediated deacetylation contributes to p53 de-stabilization. Accordingly, blocking SIRT1 in prostate cancer is reported to stabilize p53 leading to the inhibition of glycolysis [26]. Further, SIRT1 potentiates PGC-1α transcriptional activity in promoting mitochondrial biogenesis and oxidative phosphorylation [21]. With the rational that irradiation elevates the energy needs of a cell to enable DNA and organelle repair for cell recovery [27], targeting metabolic pathways could mediate radiation resistance. We tested the role CD105 has on the acute effects on DNA damage repair as well as its chronic energy needs downstream of a new target, SIRT1, in the context of irradiation.

## Results

### CD105 expression in prostate cancer upon radiation

**Fig. 1:**
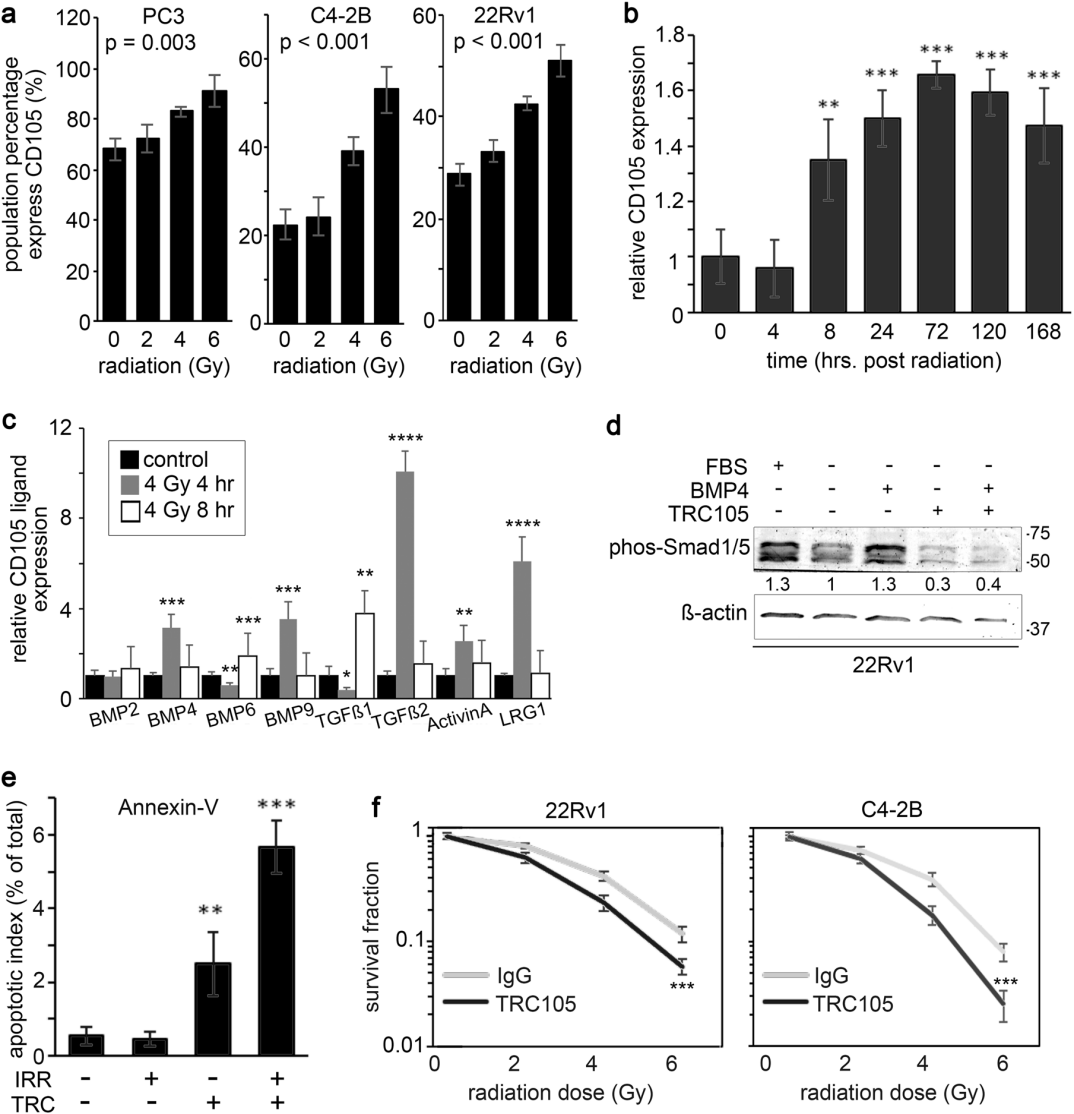
Radiation-induced CD105 expression in prostate cancer cells supports radio-resistance. **a** Cell surface CD105 expression was measured in cell lines at 72 h following a dose range of irradiation (0, 2, 4, or 6 Gy). **b** The durability of cell surface CD105 expression in 22Rv1 was determined 0, 0.5, 4, 8, 24, 48, 72, 120, and 168 h following 4 Gy irradiation. CD105 cell surface expression fold change was normalized to levels expressed prior to irradiation. **c** The mRNA expression of CD105 ligands was measured at 0, 4, and 8 h post radiation by rtPCR. Expression was normalized to GAPDH and to the 0 h time point. **d** Western blot for phosphorylated Smad1/5 was measured in 22Rv1 cells in the presence or absence of serum starvation and treatment with 50 ng/ml BMP4 or TRC105. β-actin expression served as the loading control. Molecular weight (kDa) is indicated. **e** Annexin-V expression was measured in 22Rv1 cells by FACS analysis 5 days following 4 Gy irradiation and treatment of IgG or TRC105. **f** Clonogenic assay was measured 10 days following irradiation of 22Rv1 and C4-2B cells in a dose range of 0–6 Gy in the presence of IgG or TRC105. Data are reported as a mean ± S.D. of three independent experiments (*p < 0.05, **p < 0.01, ***p < 0.001 compared to control, unless otherwise indicated)

CD105 is implicated in resistance to therapy in several cancers, including ovarian, gastric, and breast cancer [13, 28, 29]. Fluorescence-activated cell sorting (FACS) analysis revealed that prostate cancer cell lines, PC3, C4-2B, and 22Rv1, upregulate cell surface CD105 expression when exposed to irradiation (Fig. 1a). Expression of cell surface CD105 was both radiation dose- and time-dependent (Fig. 1b). While 2 Gy radiation did not significantly upregulate CD105 expression, doses of 4 and 6 Gy significantly increased CD105 for all three cell lines. Further, CD105 expression in 22Rv1 showed a significant elevation by 8 h after 4 Gy radiation that persisted for at least 1 week. As CD105 can facilitate signaling by interacting with a number of different ligands, we next tested for the expression of a panel of TGF-β/BMP ligands post radiation. We found a significant elevation of BMP4, BMP6, BMP9, TGF-β1, TGF-β2, Activin A, and LRG1 by irradiation (Fig. 1c).

Next, we sought to identify the role of CD105/BMP signaling in prostate cancer radiation response by blocking BMP-dependent CD105 signaling using TRC105. To confirm the ability of TRC105 in modulating BMP signaling, we analyzed phosphorylation of SMAD1/5 and the expression of *ID1*, a BMP target gene, in 22Rv1 stimulated with BMP4 under serum-free conditions (Fig. 1d, Supplemental Fig. 1). A known BMP antagonist, noggin, was used to confirm BMP-dependent regulation of *ID1* expression by TRC105. Importantly, TRC105 did not affect TGF-β-dependent expression of *COL1A1*, while the TGF-β inhibitor LY-364947 effectively inhibited TGF-β induction of *COL1A1* (Supplemental Fig. 1). Combining TRC105 with radiation significantly increased apoptosis as measured by cell surface Annexin-V expression, compared to radiation alone (*p* value < 0.01, Fig. 1e). To determine if CD105 confers radio-resistance, clonogenic survival assays were performed comparing IgG- or TRC105-treated 22Rv1 and C4-2B cell lines with increasing doses of radiation (Fig. 1f). In both these cell lines, treatment with TRC105 sensitized prostate cancer cells to radiation (*p* value < 0.001). Together, radiation-induced CD105 seemed to regulate prostate epithelial cell death and clonogenicity.

### Radiation-induced BMP mediates SIRT1-dependent DNA damage repair

**Fig. 2:**
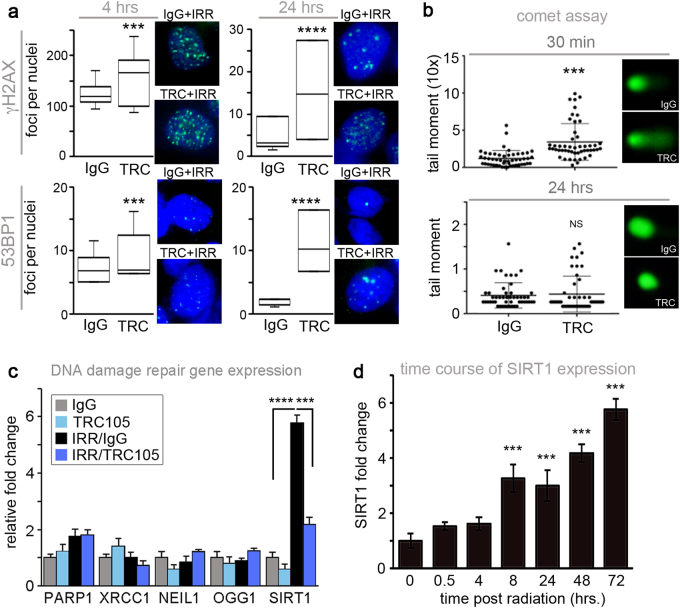
TRC105 induces transient DNA damage and repression of SIRT1 induction. 22Rv1 were pre-treated with IgG or TRC105 24 h prior to irradiation with 4 Gy. **a** γ-H2AX or 53BP1 were immunolocalized at 4 and 24 h post irradiation. Foci per nuclei were quantified (*n* = 100). Representative images are shown for γ-H2AX (green) and 53BP1 (green) foci, nuclear counterstained with DAPI (blue). **b** Comet assay was performed 30 min and 24 h following irradiation. The tail moment was quantified (*n* = 50). **c** The mRNA expression of DNA damage repair genes were measured by rtPCR 72 h post irradiation. **d** *SIRT1* mRNA expression was measured in a time course 0–72 h following 4 Gy irradiation of 22Rv1. *SIRT1* mRNA expression was normalized to *GAPDH* and to untreated. Data are reported as a mean ± S.D. of three independent experiments (***p < 0.001, ****p < 0.0001, NS not significant, compared to control)

The upregulation of CD105 by irradiation and its potential consequence on cell death suggested that CD105 may be involved in the DNA damage response. To test if impaired of DNA damage repair is the mechanism by which TRC105 conferred radio-sensitivity, γ-H2AX and p53-binding protein (53BP1) foci quantitated following 4 Gy radiation. Combined, irradiation and TRC105 treatment resulted in a significant elevation in γ-H2AX and 53BP1 foci 4–24 h post irradiation, compared to irradiation and IgG control (Fig. 2a). However, by 48 h post irradiation, there were no significant differences in DNA double-stranded breaks between the experimental groups (data not shown). The alkaline comet assay provided a measure of single-stranded DNA breaks induced by irradiation in the presence and absence of TRC105. There was a significant increase in tail moment of TRC105-treated cells 30 min following irradiation compared to radiation plus IgG (*p* value < 0.001), but there was no difference between the two groups after 24 h (Fig. 2b). Antagonizing BMP signaling downstream of CD105 by TRC105 administration impaired the repair of both double- and single-stranded DNA damage mediated by irradiation, to suggest CD105 as a target for radiation sensitivity.

To better understand the observed longer-term effects of radiation in the presence of TRC105, we examined the expression of DNA repair genes following 4 Gy irradiation. As expected, double-stranded DNA repair genes (*PARP1* and *XRCC1*) and the base excision repair genes (*NEIL1* and *OGG1*) were back to baseline levels 72 h following irradiation as most DNA damage had been repaired (Fig. 2c). Interestingly, *SIRT1*, a critical DNA damage repair component with deacetylase activity was found to be elevated approximately sixfold by irradiation over control (*p* value < 0.0001) and nearly restored to control levels by TRC105 72 h after irradiation (Fig. 2c). Similar SIRT1 mRNA expression patterns were observed with C4-2B cells (Supplemental Fig. 2a). *SIRT1* was found to be significantly upregulated in a time-dependent and radiation dose-dependent manner in both 22Rv1 and C4-2B cells at the mRNA and protein levels (Fig. 2d and Supplemental Fig. 2b-e). Unlike the other DNA damage repair genes that are acutely active following irradiation, *SIRT1* is chronically expressed in irradiated prostate cancer cells.

**Fig. 3:**
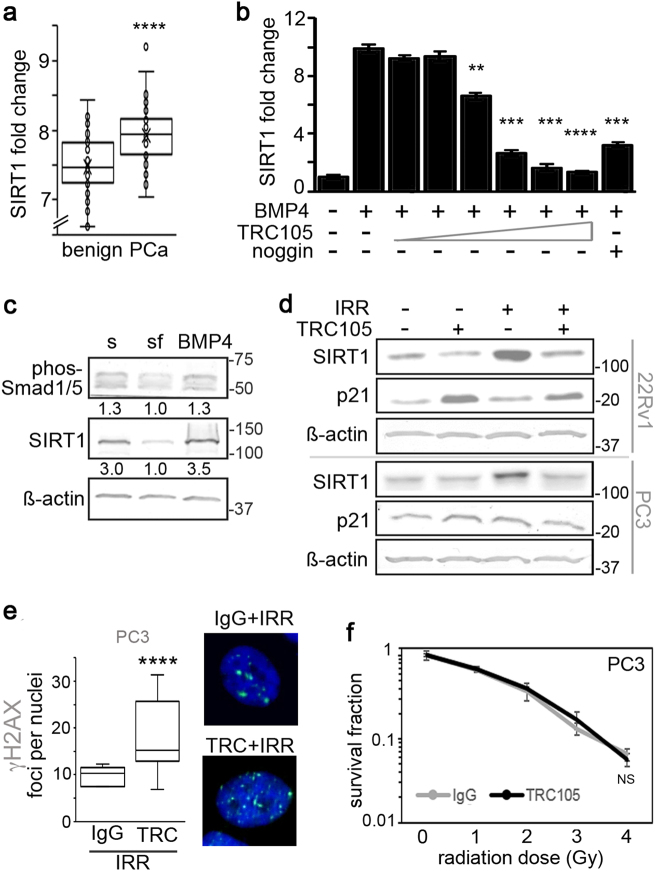
Radiation induces BMP-mediated *SIRT1* expression. **a** Fold change of *SIRT1* mRNA in benign prostate and prostate cancer patients, obtained from R2-Genomics analysis is expressed (*n* = 95). **b** *SIRT1* mRNA expression was measured in 22Rv1 under serum-free conditions with 50 ng/ml BMP4, in the context of increasing doses of TRC105 (0.05, 0.1, 0.5, 1, 5, or 10 µg/ml) or with 50 ng/ml noggin. *SIRT1* mRNA expression was normalized to GAPDH and to serum-treated control. **c** Western blot for phosphorylated Smad1/5, SIRT1, and β-actin expression was measured in 22Rv1 cells following serum starvation and treatment with 50 ng/ml BMP4. Densitometric quantitation is indicated under each band. **d** SIRT1 and p21 protein expression was measured 72 h after irradiating (4 Gy) 22Rv1 and PC3 cells in the presence of IgG or TRC105 prior to irradiation. **e** γ-H2AX (green) was immunolocalized in PC3 cells following treatment with IgG, TRC105, and 4 Gy irradation at 4 h. Cells were nuclear counterstained with DAPI (blue). Foci per nuclei were quantified (*n* = 100) (****p < 0.0001 as compared to control). **f** Clonogenic survival assay was performed on p53-null PC3 cells at indicated doses of radiation. No significant (NS) radiation sensitization was had with TRC105 compared to IgG control. mRNA expression are reported as a mean ± S.D. (**p < 0.01, ***p < 0.001, ****p < 0.0001, compared to control)

The role of SIRT1 in tumors has long been contentious as it has been shown to act as both a tumor suppressor as well as tumor promoter [23]. Therefore, we sought to compare *SIRT1* levels in patient samples to determine its role in prostate cancer. Using R2-Genomics analysis, we compared SIRT1 expression in patient samples from the German Cancer Research Center and National Center of Tumor Diseases Affymetrix GeneChip exon array dataset with benign tissue (*n* = 48) and prostate cancer tissue (*n* = 47) [30]. The comparison validated SIRT1 expression was significantly upregulated in prostate cancer samples (*p* value < 0.0001, Fig. 3a). Since blocking CD105/BMP signaling with TRC105 resulted in limiting radiation-induced SIRT1 expression, we investigated the role of BMP4 on SIRT1 expression. We found antagonizing CD105 with TRC105 effectively blocked BMP4-dependent induction of SIRT1 in a TRC105 dose-dependent manner, similar to that mediated by noggin (Fig. 3b). Strikingly, the BMP4-induced SIRT1 protein expression in serum-starved 22Rv1 associated with phosphorylated SMAD1/5 (Fig. 3c), suggesting a role for CD105 in the regulation of *SIRT1* via canonical BMP signaling.

*SIRT1* expression is a known result of radiation treatment. We found that CD105/BMP signaling is necessary and sufficient for SIRT1 expression downstream of radiation treatment, as TRC105 limited radiation-induced SIRT1 protein expression (Fig. 3d). Interestingly, both 22Rv1 and p53-null PC3 cells similarly induced SIRT1 in a CD105-dependent manner. However, the lack of p53 in PC3 disabled p21 induction by TRC105 treatment, observed in 22Rv1 cells. This suggested CD105/BMP inhibition of p21 was p53-dependent. However, SIRT1 is known to de-stabilize p53 by de-acetylating p53-K382. Thus, to test *SIRT1* function downstream of CD105, we measured p53 regulation by immuno-precipitation and immunoblotting for acetylated p53-K382 following treatment with either TRC105 or nicotinamide, an inhibitor of SIRT1 activity, in the context of radiation. As expected, radiation alone resulted in increased total p53 expression compared to control. Inhibiting SIRT1 expression with TRC105 resulted in elevated p53 acetylation and total p53 expression, compared to radiation alone (Supplemental Fig. 3a). Irradiation-induced CD105 mediated SIRT1 expression and function as revealed by suppressed p53 expression.

The apparent SIRT1 regulation of p53 by CD105/BMP signaling prompted us to test the efficacy of TRC105-mediated radiation sensitization in PC3 cells. Yet, in PC3 cells, TRC105 caused significantly elevated DNA double-stranded breaks following irradiation, compared to IgG control (Fig. 3e). However, when we knocked down p53 in 22Rv1 (siP53), TRC105 did not further γ-H2AX foci numbers significantly compared to scrambled siRNA or IgG controls (Supplemental Fig. 3b, c). Accordingly, in clonogenic assays treating PC3 with TRC105 at increasing doses of radiation did not provide radiation sensitization over IgG control (Fig. 3f). PC3 cells have previously been described as unresponsive to SIRT1 inhibitors [24]. While loss-of-function p53 mutations are rare in prostate cancer, 50–75% of pancreatic cancers have p53 mutations [31, 32]. We therefore tested two p53 mutant pancreatic cancer cell lines, MIAPACA-2 and HPAF-II, for radiation responsiveness in the context of TRC105 treatment. In validating the findings with PC3 cells, neither MIAPACA-2 nor HPAF-II were sensitized to radiation by CD105 antagonism (Supplemental Fig. 3d, e). This suggested the novel CD105-SIRT1 signaling axis requires p53 for radiation responsiveness.

### PGC-1α and cellular energy production are regulated by CD105/BMP

**Fig. 4:**
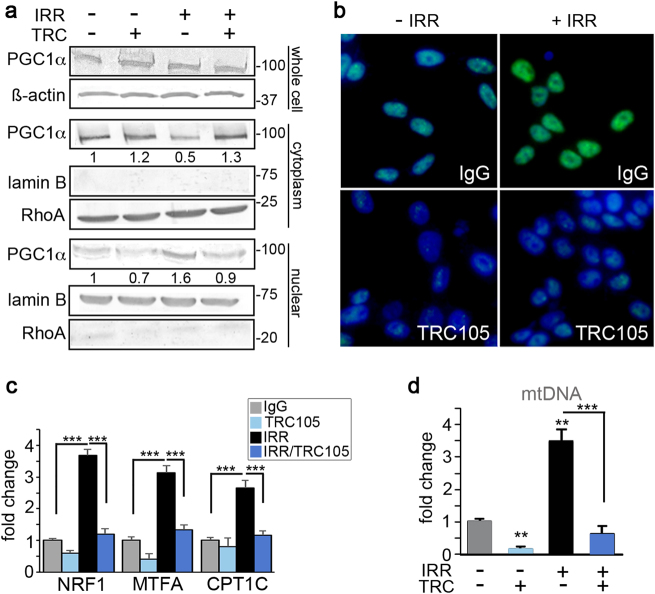
PGC-1α and mitochondrial biogenesis are regulated by CD105/BMP. 22Rv1 cells were incubated with IgG or TRC105 with or without 4 Gy irradiation. All measurements were made 72 h post irradiation. **a** Western blot for whole-cell lysate, nuclear, and cytoplasmic fractions were independently analyzed for PGC-1α expression. Loading controls included β-actin (whole cell), lamin B (nuclear marker), and Rho A (cytoplasm marker). Molecular weights (kDa) of the ladder are indicated. **b** Immunofluorescent localization of PGC-1α (green) was visualized with DAPI (blue) nuclear counterstain. **c** The mRNA expression of PGC-1α target genes, *NRF1*, *MTFA*, and *CPT1C* were measured. MRNA expression was normalized to *GAPDH* and untreated. **d** Mitochondrial DNA (mtDNA) was measured from total DNA extracts and normalized to nuclear DNA and to untreated. Data are reported as means ± S.D. of three independent experiments (**p < 0.01, ***p < 0.001, compared to control unless otherwise indicated)

Cell recovery from radiation-induced damage requires large amounts of energy. Further, radio-resistant cancer cells have been shown to induce mitochondrial content and mitochondrial DNA (mtDNA) accumulation in response to radiation [33]. Therefore, we reasoned that targeting cellular metabolism may play a role in the radiation sensitization seen with TRC105 and its inhibition of radiation-induced SIRT1 upregulation. We tested another downstream function of SIRT1, the activation of PGC-1α, a transcription factor involved in mitochondrial biogenesis. Activation and nuclear localization of PGC-1α requires deacetylation by SIRT1 [21]. The treatment of 22Rv1 cells with 4 Gy radiation in the presence of IgG or TRC105 had no effect on PGC-1α expression, by western blotting of the whole-cell lysate (Fig. 4a). However, closer examination of subcellular localization through organelle fractionation demonstrated PGC-1α depletion from the cytoplasmic fraction and accumulation in the nuclear fraction in the context of radiation. Blocking CD105 prevented radiation-induced nuclear translocation of PGC-1α. Immunofluorescent localization corroborated these same findings (Fig. 4b). PGC-1α target genes involved in oxidative stress, mitochondrial biogenesis, and fatty acid oxidation: *NRF1*, *MTFA*, and *CPT1C*, respectively, were significantly elevated by radiation (*p* value < 0.001, Fig. 4c). The same genes were significantly downregulated by the added treatment with TRC105 in both 22Rv1 and C4-2B cells (Fig. 4c and Supplemental Fig. 4a). Consequently, mtDNA content was significantly elevated by irradiation (*p* value < 0.0001), to be restored to control levels by antagonizing CD105 (Fig. 4d). The evaluation of specific mitochondrial electron transport chain proteins showed TRC105 treatment downregulated complex I-NDUF88 and complex IV-MTCO1 (Supplemental Fig. 4b). To further validate the importance of PGC-1α in TRC105-mediated radiation sensitization, we knocked down PGC-1α in 22Rv1 and measured γ-H2AX (Supplemental Fig. 4c). Silencing of PGC-1α resulted in a significant increase in radiation-induced γ-H2AX foci per nuclei, indicating mitochondrial biogenesis was necessary for DNA damage repair. Together, we found that CD105 regulation of *SIRT1* expression affected both DNA damage and maintenance of mitochondrial integrity through PGC-1α in the context of irradiation.

**Fig. 5:**
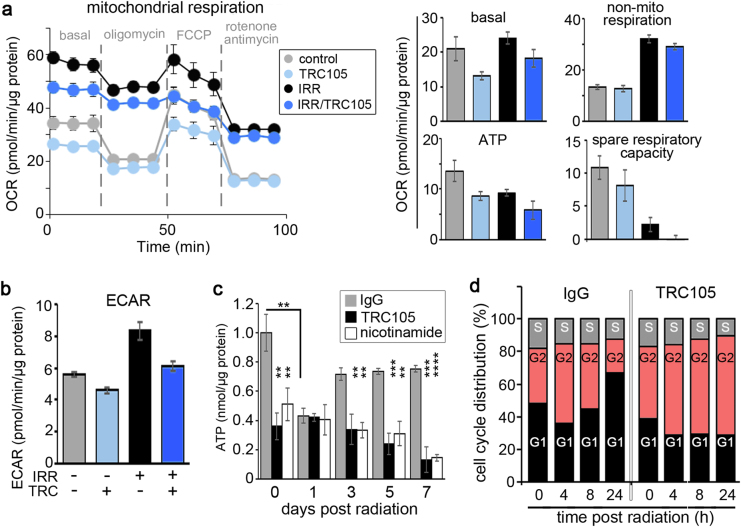
Metabolic changes induced by CD105 antagonism. **a**, **b** Cells were analyzed for mitochondrial activity 168 h following 4 Gy irradiation in the presence of IgG or TRC105. **a** Basal respiration, non-mitochondrial respiration, mitochondrial ATP, spare respiratory capacity, and **b** extracellular acidification rate were quantitated. Data are reported as mean ± S.D. of a representative experiment (*n* = 5) of three independent experiments. **c** Total cellular ATP was measured 0, 24, 72, 120, and 168 h following 4 Gy irradiation in the context of IgG, TRC105, or nicotinamide treatment. Data are reported as mean ± S.D. of three independent experiments (**p < 0.01, ***p < 0.001, ****p < 0.0001). **d** Cell cycle analysis was performed on 22Rv1 at 0, 4, 8, and 24 h post irradiation in the presence of IgG or TRC105 (*n* = 3) in three independent experiments

Since PGC-1α was crucial for DNA damage repair, we studied the functionality of the mitochondria after radiation and TRC105 treatment through the measurement of oxygen consumption rates (OCRs) as an indicator of oxidative phosphorylation activity. Radiation treatment elevated non-mitochondrial respiration compared to cells not irradiated, regardless of TRC105 treatment (Fig. 5a). However, when comparing only mitochondrial respiration, the basal oxygen consumption of irradiated to non-irradiated cells was similar. Not surprisingly, radiation-mediated mitochondrial damage manifested in decreased ATP production and a depletion of spare respiratory capacity. Antagonizing CD105 in the context of radiation resulted in a decrease in basal oxidative phosphorylation, further decrease in ATP production, and spare respiratory capacity compared to radiation alone. Mitochondrial ATP production downregulated by irradiation was found to increase reliance on glycolysis, as measured by extracellular acidification rate (ECAR), in 22Rv1 cells (Fig. 5b). But, further addition of TRC105 inhibited glycolysis in 22Rv1 cells.

Glycolysis and oxidative phosphorylation are crucial for energy production in the form of ATP. We found a significant depletion of cellular ATP stores within 1 day of radiation treatment (*p* value < 0.01), which seemed to be restored to levels close to control by 3 days in 22Rv1 cells (Fig. 5c). When *SIRT1* expression was inhibited by CD105 antagonism or its function with nicotinamide, cellular ATP stores were significantly lower than the non-irradiated control and further depleted by irradiation. The reliance of intact p53 for TRC105 radio-sensitization suggested inhibition of glycolysis by p53 is critical to radio-sensitization [34, 35]. To independently test the consequence of ATP derived from oxidative phosphorylation on cell proliferation, 22Rv1, siP53 22Rv1, and p53-null PC3 cells were treated with oligomycin, an ATP synthase inhibitor. 22Rv1 proliferation limited by irradiation was further downregulated by oligomycin (*p* value < 0.01, Supplemental Fig. 5). In contrast, the PC3 cells, had reduced cell counts with irradiation by about 50%, but were insensitive to inhibition of mitochondrial ATP synthesis. Silencing of p53 resulted in reduced susceptibility of 22Rv1 to oligomycin treatment than scrambled siRNA. Silencing of p53 was not as robust as PC3 response to oligomycin, possibly due to incomplete silencing or long-term metabolic adaptation of p53 loss in PC3. Hence, the p53 response is important for radiation-induced maintenance of energy homeostasis and cell division.

The impact of radiation on the cell cycle is well described as causing a G2 cell cycle arrest followed by cell cycle redistribution. In view of the fact that both p53-dependent p21 activity and mitochondrial dysregulation can similarly impact G2 cell cycle arrest [36–38], we found that irradiating 22Rv1 cells, in the presence of IgG, caused an accumulation of cells in G2 phase by 4 h, to then recover to control levels by 8 h (Fig. 5d). Interestingly, TRC105 alone expanded the G2 cell population. The combination of radiation and TRC105 treatment resulted in G2 cell cycle arrest that did not resolve by 24 h. Therefore, radiation-induced CD105 signaling helps restore metabolic activity chronically to enable the G2/M cell cycle transition.

### Antagonizing CD105 confers radio-sensitivity in vivo

**Fig. 6:**
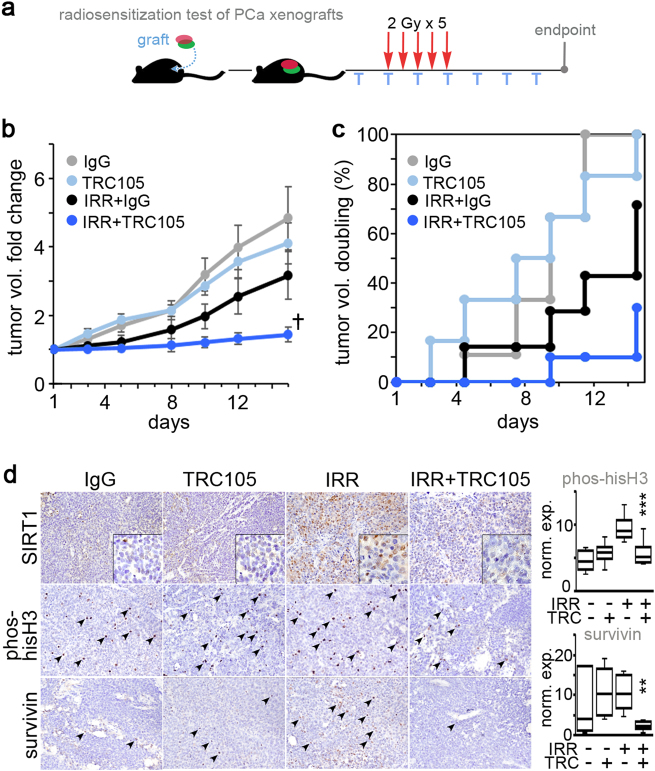
Antagonizing CD105 confers radio-sensitivity in vivo. **a** Tumor volumes were longitudinally measured. When tumor average volume reached 80 mm^3^ mice were treated with IgG or TRC105 (T) in the context of radiation (2 Gy for 5 days). Tumors were harvested 15 days after the first dose of radiation (*n* = 6). **b** Tumor volume fold change was normalized to the first dose of radiation (^†^*p* = 1 × 10^−9^). Each treatment was compared for doubling of tumor volume as a function of time as depicted in the cumulative incidence plot. **d** Histochemical localization of SIRT1, phosphorylated histone H3, and survivin was performed on paraffin-embedded tumor tissues. Phosphorylated histone H3 and survivin expression was quantitated as a measure of total cells per field per tumor (**p < 0.01, ***p < 0.001)

Lastly, we tried to determine the role of CD105 on radio-resistance using a 22Rv1 xenograft model. Mice engrafted with 22Rv1 were given one dose of IgG or TRC105 72 h prior to irradiation when the tumor reached 0.8 cm^3^. The tumors were irradiated (2 Gy) for 5 consecutive days and TRC105 was administered 3 times a week for the duration of the treatment schedule (Fig. 6a). We found TRC105 alone did not influence tumor volume compared to the control IgG treated group (Fig. 6b). The tumor volumes for the irradiated IgG group was significantly lower a week after irradiation compared to control, but by 2 weeks this group was not significantly different from the non-irradiated groups. Conversely, the combination of radiation- and TRC105-treated tumor volume was dramatically lower than the other three experimental groups (repeated measures analysis of variance (R-ANOVA) *p* value = 1 × 10^−9^ and *F*-statistic of 11.4). The tumor-doubling time was appreciably reduced by combining TRC105 with irradiation compared to either treatment alone. Immunohistochemical staining of the tumors showed a radiation-induced increase in SIRT1, abrogated by the treatment with TRC105 (Fig. 6c). The mitotic index measurement by phosphorylated histone H3 quantitation indicated a significant downregulation by the combination of TRC105 and irradiation (*p* value = 0.0002). Concomitantly, the expression of survivin, an anti-apoptotic protein, was also markedly decreased in irradiated tumors treated with TRC105 (*p* value = 0.002). Thus, mitigating radiation-elevated CD105-induced *SIRT1* by TRC105 is an effective radiation sensitizer for p53-intact prostate cancer.

## Discussion

**Fig. 7:**
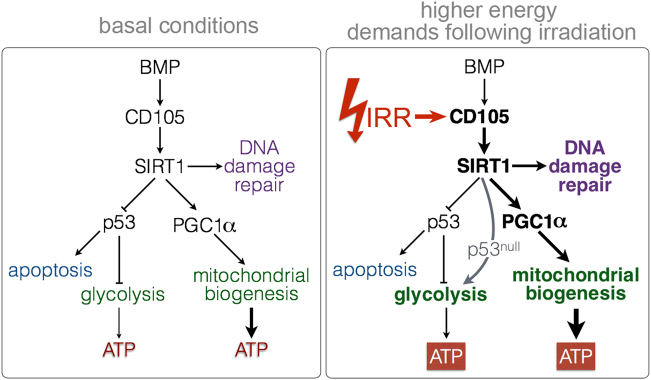
A Schematic depiction of the mechanism of CD105/BMP signaling in conferring radiation resistance. Radiation of prostate cancer results in upregulation of cell surface CD105 expression. The ensuing CD105/BMP signaling is essential and necessary for SIRT1 expression and downstream activity. SIRT1 facilitates DNA damage repair, destabilizes p53, and activates of PGC-1α in response to radiation. Consequently, CD105 can regulate glycolysis and mitochondrial biogenesis to meet the higher energy demands necessary for survival following irradiation. The loss of functional p53 enables glycolysis as a source of ATP generation and survival

Our work demonstrates the role of CD105 upregulation in response to irradiation. Most of what is known about CD105 signaling has been elucidated from studying endothelial cells and vascular diseases characterized by CD105 mutations such as hereditary hemorrhagic telangiectasia and preeclampsia. In endothelia, CD105 expression is tightly regulated by hypoxia inducible factor-1 alpha (HIF-1α) [39]. Since radiation induces HIF-1α, an increase in CD105 expression with radiation treatment was anticipated [40]. Instead of performing studies where CD105 is silenced or knocked out, as others have done in the context of DNA-damaging chemotherapy studies [13], we deliberately used a neutralizing antibody that inhibited the CD105/BMP signaling axis without affecting TGF-β signaling. In breast cancer, radiation causes an increase in serum TGF-β levels and inhibiting tumor TGF-β signaling can sensitize to radiation [15, 41, 42]. The mechanism of TGF-β inhibition-associated radiation sensitization is primarily associated with impaired DNA damage repair [42]. Thus, knocking out CD105 would achieve BMP signaling inhibition, but would activate TGF-β signaling—not a desired outcome. We found inhibiting BMP signaling through TRC105 or noggin could inhibit SIRT1 expression. We identified a new role for CD105 in mediating metabolic adaptations to stress caused by radiation through the regulation of a novel CD105/BMP target, SIRT1 (Fig. 7). SIRT1 inhibitors have been effective in sensitizing a variety of cancer cell lines to DNA-damaging agents, including radiation. However, the mechanism by which SIRT1 inhibitors, such as nicotinamide, sensitize cancers to therapy has largely been attributed to SIRT1’s role in DNA damage repair. We show that suppressing SIRT1 expression by antagonizing CD105/BMP signaling leads to increased DNA damage with radiation acutely (Fig. 2), exacerbated by severe depletion of energy chronically (Fig. 5).

The induction of CD105 by radiation in the tumor epithelia is exploited by the administration of TRC105. As a tool to interrogate the mechanism of action, TRC105 demonstrated that effective radiation sensitizers do not necessary need to solely act as DNA damage effectors. In addition to the capacity of the BMP-CD105-SIRT1 signaling axis to support DNA repair downstream of p53, we demonstrated it conferred radio-resistance through the induction of mitochondrial biogenesis through PGC-1α activity. TRC105, as a single agent, has little impact on cell proliferation despite its inhibitory effects on mitochondrial biogenesis and glycolysis, as the energy needs of a non-irradiated cells are minimal. However, the added ATP requirement to repair radiation-induced damage makes mitochondrial biogenesis obligatory following irradiation. Prostate cancer cells respond to irradiation by increasing SIRT1 expression (Fig. 2) to transiently increase glycolysis, by stabilizing p53 (Fig. 3), and mitochondrial biogenesis, by potentiating PGC-1α activity (Fig. 4), to sustain immediate and long-term energy requirements (Fig. 5) [20, 34]. Antagonizing CD105 with TRC105 acts by acutely limiting DNA damage repair and chronically preventing recovery by depleting their energy stores, thereby limiting prostate cancer expansion. PARP1 inhibitors have been effective radio-sensitizers for prostate and other cancers by furthering DNA damage accumulation. Unlike for PARP1 inhibitors, intact p53 function was necessary for TRC105-mediated radio-sensitization. TRC105 exploits the metabolic addiction induced by radiation in p53-intact cells. Addressing the CD105-mediated radiation resistance mechanism with TRC105 can serve as a synthetic lethal strategy for patients on radiotherapy.

## Materials and Methods

### Cell lines and culture

CWR22Rv1 (22Rv1), PC3, C4-2B, MIAPACA-2, and HPAF-II cells were purchased (American Type Culture Collection). Cell lines were cultured in RPMI-1640 medium in 10% fetal bovine serum. For counting cells for proliferation, 25 000 cells/24 wells were treated with oligomycin 1 h before radiation. Cells were collected and counted using a hemocytometer 72 h post treatment using 5 wells per treatment. The Gammacell 40 Exactor (Best Theratronics, Ottawa, CA) was used for irradiation at indicated doses. 22Rv1 were silenced using pooled target-specific 19- to 25-nucleotide siRNAs by transfecting with either control siRNA-A (sc-37007, Santa Cruz Biotechnology), PGC-1α siRNA (sc-38884, Santa Cruz Biotechnology), or P53 siRNA (sc-29435, Santa Cruz Biotechnology). Cells were transfected using Lipofectamine 3000 Reagent (ThermoFisher) according to the manufacturer’s protocol.

### Reagents

TRC105 was provided from TRACON Pharmaceuticals, Inc. Cells were treated with TRC105 at a concentration of 1 µg/ml, unless noted otherwise. BMP4 (PHC9534, Gibco) and noggin (120-10 C, Peprotech) were used at 50 ng/ml. TGF-β was used at 5 ng/ml and LY-364947 was used at 10 µM. Nicotinamide was used at 200 µg/ml. Oligomycin (495455, EMD Milipore) was used at 1, 1.5, 2, and 2.5 µM concentrations for proliferation assays.

### Clonogenic survival assay

Cells were seeded 2 h prior to irradiation and treated with either IgG or TRC105. Cells were grown for 7–10 days to allow for colony formation and then fixed and stained with crystal violet in methanol. Colonies were delineated as <50 cells. Survival fraction was calculated as the ratio of the number of colonies formed to the number of colonies seeded times the plating efficiency [43].

### Immunofluorescence

Cells grown on coverslips were fixed with 4% paraformaldehyde at room temperature followed by phosphate-buffered saline (PBS) rinses. Cells were permeabilized and blocked with 0.1% Triton X-100 and 1% bovine serum albumin in PBS for 1 h at room temperature, followed by incubation with γ-H2AX (05-636, EMD Millipore), 53BP1 (SC-22760, Santa Cruz Biotechnology), or PGC-1α (SC-13067, Santa Cruz Biotechnology) antibodies at 4 °C. Alexa 488 anti-mouse and Alexa 488 anti-rabbit (Life Technologies) secondary antibodies were used at room temperature. Coverslips were mounted with Vectashield Hardset Antifade Mounting Medium with DAPI (H-1400, Vector Laboratories). Images were taken with Olympus FSX-100 and quantitated as foci per nuclei using ImageJ.

### Alkaline Comet assay

Cells were collected at indicated time points and re-suspended in low-melting-point agarose provided by Cell Biolabs’s COMET Assay kit (STA-351, Cell Biolabs). The assay was run per the manufacture’s protocol. Images were taken using an Olympus FSX-100 microscope and quantitated using the OpenComet plugin for ImageJ.

### FACS analysis

FACS experiments were performed with anti-human CD105-APC (17-1057-41, e-Biosciences) and anti-human Annexin-V-PE (BDB556422, BD Biosciences). Cell cycle was analyzed as previously reported [44]. All events were acquired on a BD Accuri C6 Plus flow cytometer and analyzed by FlowJo software v10.2.

### Protein analysis

Whole-lysate western blots were probed for the following antibodies phos-SMAD1/5 (9516, Cell Signaling Technologies), SIRT1 (9475, Cell Signaling Technologies), p21 (4060, Cell Signaling Technologies), PGC-1α (ST1202, EMD Millipore), Total OXPHOS Rodent (ab110413, Abcam), PGC-1α (ST1202, EMD Millipore), lamin B (sc-6217, Santa Cruz Biotechnology), Rho A (sc-418, Santa Cruz Biotechnology), p53 (sc-126, Santa Cruz Biotechnology), K382 acetyl-p53 (2525, Cell Signaling Technologies), and β-actin (sc-47778, Santa Cruz Biotechnology). The NE-PER Nuclear and Cytoplasmic Extraction Reagent Kit (PI-78833, Thermo Scientific) was used according to protocol. To enrich for p53, samples were immunoprecipitated using p53 N-term-Trap (pta-20, Chromotek) according to the manufacter’s protocol with addition of 200 µg/ml of nicotinamide to lysis and wash buffers.

### Quantitative real-time PCR

RNA was extracted using the RNeasy mini kit (74106, Qiagen Inc.) according to the manufacturer’s protocol. Reverse transcription and quantitative real-time PCR data were calculated by ΔΔCt method and represented relative to 18S rRNA expression. mtDNA was quantified as previously described using MTCO2 expression normalized to genomic ACTB expression [45]. (Refer to Supplemental Table 1 for primer sequences.)

### Oxygen consumption and acidification analysis

Respirometry was conducted on 22Rv1 cells using the Seahorse XF^e^24 Extracellular Flux Analyzer (Seahorse Biosciences) 7 days after radiation treatment for real-time measurements of OCR and ECAR (as a reporter of glycolysis). Cells were seeded in XF24 cell culture plates at a density of 100 000 cells/well and assay was conducted 16 h after. Prior to performing the assay, culture media was exchanged for Seahorse XF Base media (supplemented to 10 mM glucose, 1 mM pyruvate, and 1 mM glutamine, pH 7.4) and equilibrated for 1 h at 37 °C in a non-CO_2_ incubator. Final concentration of inhibitors are as follows: 2 μM oligomycin; 1.5 μM FCCP (carbonyl cyanide 4-(trifluoromethoxy)phenylhydrazone), 1 μM antimycin A; and 1 μM rotenone (Sigma). Results were normalized to protein concentration determined by Pierce BCA Protein Assay Kit.

### ATP assay

22Rv1 cells were collected at days 0, 1, 3, 5, and 7 after radiation and pellets were frozen. ATP was quantified immediately after lysis of pellets using the ATP Determination Kit (A22066, Invitrogen) according to the manufacturer’s protocol.

### Xenograft model

22Rv1 (1 × 10^6^) were suspended in 100 µl of saline with 50% rat-tail collagen and were implanted subcutaneously into the flank of 6-week-old male athymic nude mice (Envigo, Indianapolis, IN). *N* = 6 mice were used per a condition, based on previous subcutaneous tumor experiments. When average tumor volume reached 80 mm^3^, the mice were placed into four groups (IgG alone, TRC105 alone, IgG with radiation, and TRC105 with radiation) by randomization and the first dose of TRC105 or IgG was administered. Mice were treated with either IgG or TRC105 (50 µg) three times a week, unblinded. Tumor volume was recorded three times a week with digital calipers. No animals were excluded from analysis. All animal experiments were performed in accordance with the guidelines of the Institutional Animal Care and Use Committee at Cedars-Sinai Medical Center.

### Immunohistochemistry

Paraffin-embedded tissues (5 μm thick) were subjected to immunohistochemical staining as previously reported [46]. Anti-phosphorylated histone H3 (PH-H3, 06-570, Millipore), anti-survivin (2808, Cell Signal Technologies), and anti-SIRT1 (sc-74504, Santa Cruz Biotechnology) were incubated at 4 °C overnight. Secondary antibody development was performed with Dako Cytomation EnVision + mouse or rabbit labeled polymer kits (K4001 and K4003, Dako Cytomation) and visualized using 3,3′-diaminobenzidine tetrahydrochloride substrate (K3468, Dako Cytomation). Up to five fields per tissue (*n* = 4) were quantitated with Fiji (ImageJ) using a custom-written macro. Mitotic (PH-H3) index was calculated by taking the total number of positive (brown) nuclei divided by the total number of nuclei.

### Statistical analysis

Student’s *T*-test was used to compare radiation alone to radiation with treatment. Two-way ANOVA was used to compare the effect of multiple treatment groups. The R-ANOVA in MATLAB was used to calculate the *p* values for detecting tumor size differences over time. Results were expressed as individual data points or as the mean ± S.D. *p* values of <0.05 were considered statistically significant (*p < 0.05, **p < 0.01, ***p < 0.001, ****p < 0.0001).

## Electronic supplementary material

Supplemental Figures
