# Supplementary material for: Antagonizing CD105 enhances radiation sensitivity in prostate cancer
Source: Oncogene. 2018 May 2;37(32):4385–97. doi: 10.1038/s41388-018-0278-0 (PMC6085281; doi:10.1038/s41388-018-0278-0)
Supplement: Supplementary file 1 — Supplemental Figures [file 41388_2018_278_MOESM1_ESM.pdf]

Supplemental Figures

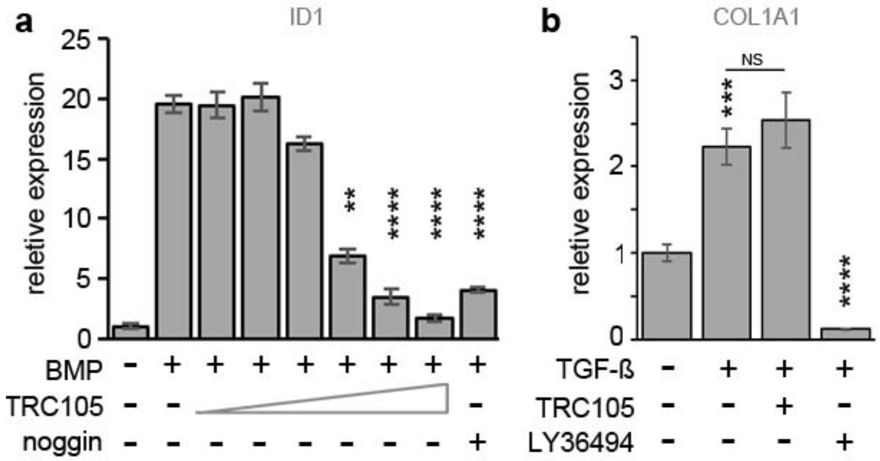

Supplemental Figure 1. a. ID1 mRNA expression was measured in 22Rv1 under serum free conditions with 50 ng/ml BMP4, IgG in the context of increasing doses of TRC105 (0.05, 0.1, 0.5, 1, 5, or 10 µg/ml). **b.** Collagen 1a1 (COL1A1) mRNA expression was measured under serum free conditions with 5 ng/ml TGF-ß, TRC105 (10 µg/ml), and/or TGF-ß antagonist (LY-36494, 10 µM). ID1 and COL1a1 mRNA expression was normalized to GAPDH. (**p<0.01, ***p<0.001, ****p<0.0001, as compared to control)

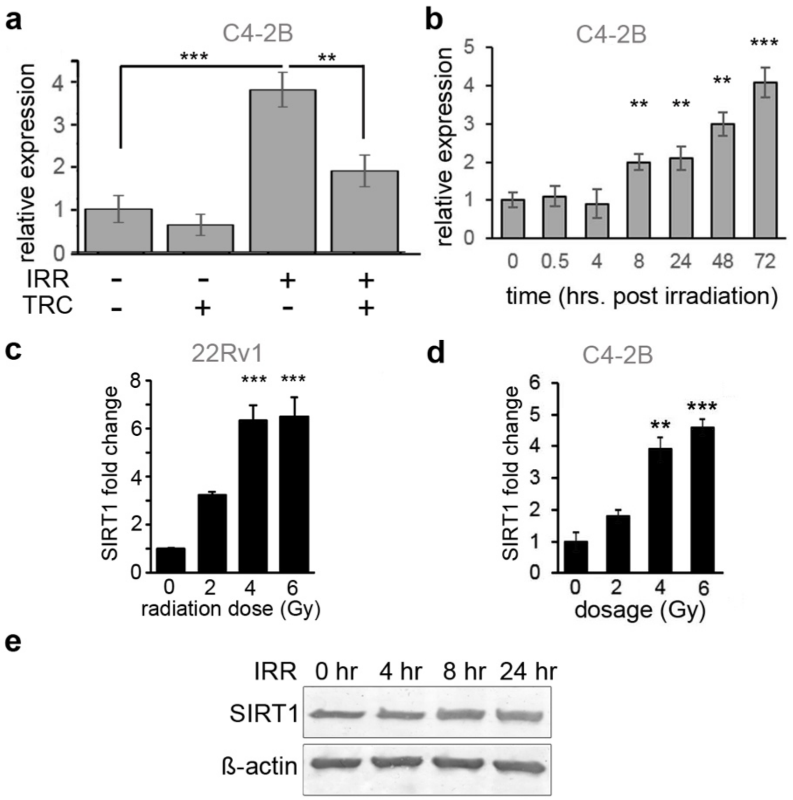

Supplemental Figure 2: SIRT1 mRNA expression was quantitated. **a.** C4-2B were pre-treated with IgG or TRC105 prior to irradiation with 4 Gy and compared for relative SIRT1 mRNA expression 72 hours after irradiation. **b.** C4-2B cells were irradiated (4 Gy) and SIRT1 expression measured 0, 0.5, 4, 8, 24, 48, and 72 hours post- irradiation. **c.** 22Rv1 were irradiated (0, 2, 4, or 6 Gy) and SIRT1 expression measured 72 hours post-irradiation. **d.** C4-2B were irradiated (0, 2, 4, or 6 Gy) and SIRT1 expression measured 72 hours post-irradiation. SIRT1 mRNA was normalized to GAPDH and to untreated. **e.** SIRT1 protein expression was measured at 0, 4, 8, 24 hours post 4Gy irradiation in 22Rv1 by western blot. Western blot was probed for SIRT1 and β-actin (**$p<0.01$, ***$p<0.001$ as compared to control).

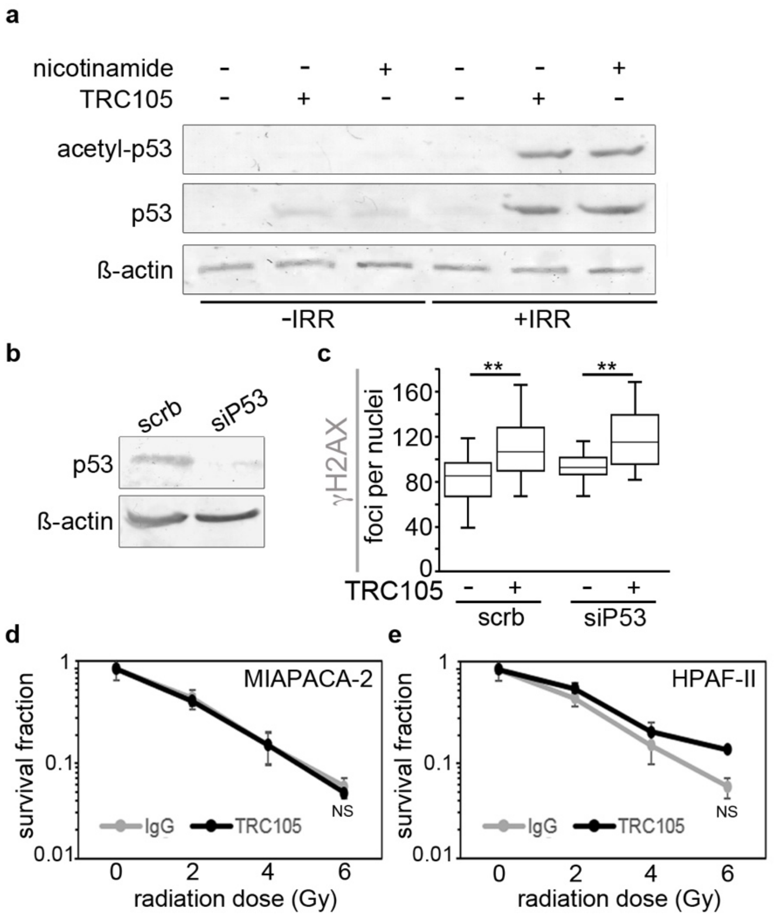

Supplemental Figure 3: a. 22Rv1 cells were Western blotted 0 or 7 days following radiation in the presence of IgG, TRC105, or nicotinamide. Whole lysate was probed for total p53 and acetylated-p53 following p53 enrichment by immunoprecipitation. 22Rv1 were transfected with either Scrambled siRNA (scrb) or p53 siRNA (siP53) 48 hours prior to irradiation or collection for western blot analysis. **b.** Western blots were probed for total p53 and β-actin to determine efficient p53 silencing. **c.** γ-H2AX was immunolocalized at 4 hours post 4 Gy irradiation of scrb or siP53 22Rv1, treated with IgG or TRC105 (**p<0.01). Foci per nuclei were quantified (n = 100). **d,e.** Clonogenic survival assays were performed on two p53 mutant pancreatic cancer cell lines, MIAPACA-2 and HPAF-II with indicated doses of radiation. No significant (NS) radiation sensitization was had with TRC105 compared to IgG control.

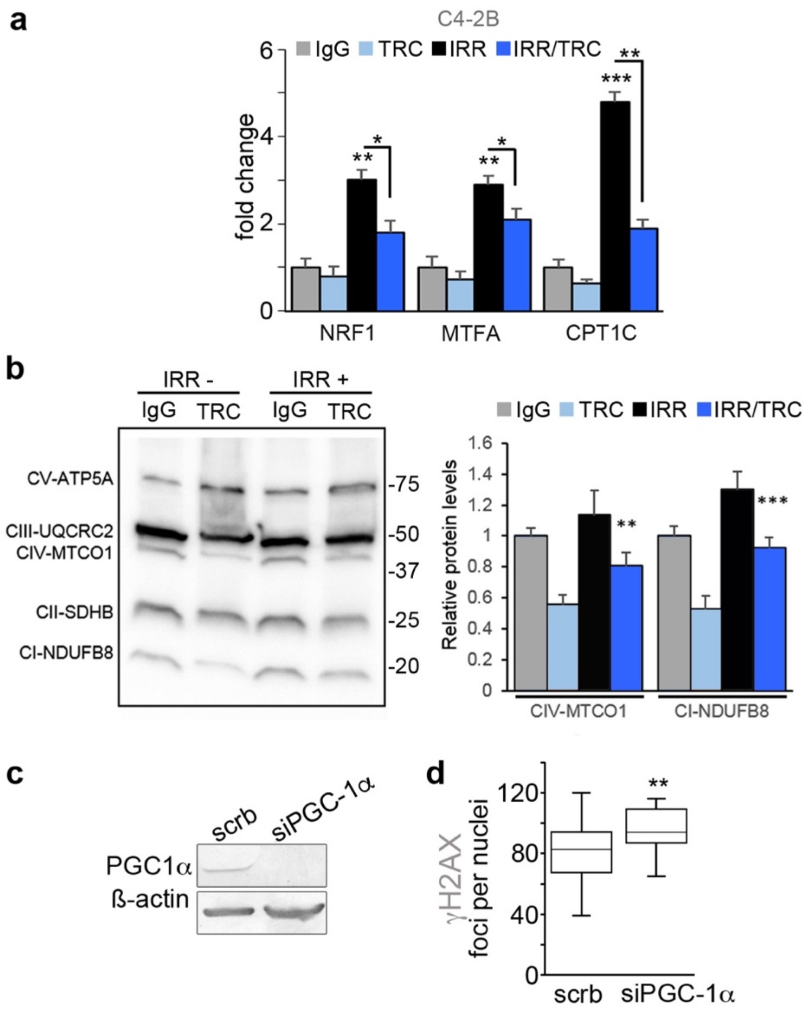

Supplemental Figure 4: a. The mRNA expression of PGC-1α target genes, NRF1, MTFA, and CPT1C were measured in C4-2B cells. MRNA expression was normalized to GAPDH and untreated. **b.** 22Rv1 were treated with IgG or TRC105 prior to irradiation with 4 Gy. Lysate was collected 72 hours post-irradiation for Western blot. Blots were probed for a cocktail of mitochondrial complex proteins. Protein levels of MTCO1 of complex-IV and NDUFB8 of complex-I in 3 independent experiments were normalized to Ponceau S. MTCO1 and NDUFB8 were significantly lower in 4 Gy with TRC105 compared to radiation alone. 22Rv1 were transfected with either scrambled siRNA (scrb) or PGC-1α siRNA (siPGC-1α) 48 hours prior to radiation treatment or collection for western blot analysis. **c.** Western blots were probed for total PGC-1α and β-actin to determine efficient PGC-1α knockdown. **d.** γ-H2AX was immunolocalized in scrb or siPGC-1α 22Rv1 cells 4 hours after 4 Gy irradiation. Foci per nuclei were quantified (n = 100). (*p<0.05, **p<0.01, ***p<0.001).

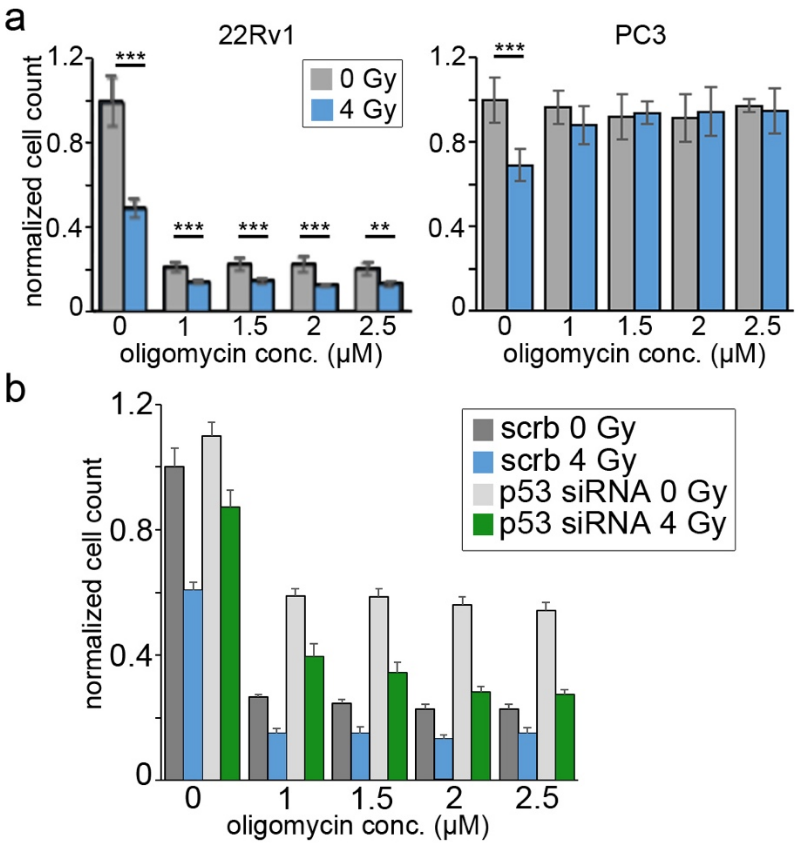

Supplemental Figure 5. a. The role of ATP depletion on radiation sensitivity was tested by treating 22Rv1 and PC3 cells with indicated doses of ATPase inhibitor, oligomycin and exposed to 4 Gy irradiation. Cell counts were performed 72 hours following irradiation. (**p<0.01, ***p<0.001). **b.** Scrambled and p53 siRNA transfected 22Rv1 were treated with indicated doses of oligomycin and exposed to 4Gy irradiation. Cell counts were performed 72 hours following irradiation.

Supplemental Table 1. Primer Sequences

Gene	Sequence
ID1 Forward	5'-AAT CAT GAA AGT CGC CAG TG-3'
ID1 Reverse	5'-ATG TCG TAG AGC AGC ACG TTT-3'
COL1A1 Forward	TCTGCGACAACGGCAAGGTG
COL1A1 Reverse	GACGCCGGTGGTTTCTTGGT
SIRT1 Forward	5'-TGC TGG CCT AAT AGA GTG GCA AAG-3'
SIRT1 Reverse	5'- GGC ATG TCC CAC TAT CAC TGT-3'
NRF1 Forward	5'-CAG CAG GTC CAT GTG GCT ACT-3'
NRF1 Reverse	5'-GCC GTT TCC GTT TCT TTC C-3'
MTFA Forward	5'-GAT GCT TAT AGG GCG GAG TGG-3'
MTFA Reverse	5'-GCT GAA CGA GGT CTT TTT GGT-3'
CPT1C Forward	5'-TTT CTG GGT GAC GGT GAT CTC-3'
CPT1C Reverse	5'-CAT ATG TCC AAT CCC AGT GCA A-3'
GAPDH Forward	5'-CAT GAG AAG TAT GAC AAC AGC CT-3'
GAPDH Reverse	5'-AGT CCT TCC ACG ATA CCA AAG T-3'
MT-CO2 Forward	5'-CCT GCG ACT CCT TGA CGT TG-3'
MT-CO2 Reverse	5'-AGC GGT GAA AGT GGT TTG GTT-3'
ACTB Forward	5'-TCA CCC ACA CTG TGC CCA TCT ACG A-3'
ACTB Reverse	5'-CAG CGG AAC CGC TCA TTG CCA ATG G-3'
BMP2 Forward	5'-ATGGATTCGTGGTGGAAGTG-3'
BMP2 Reverse	5'-GTGGAGTTCAGATGATCAGC-3'
BMP4 Forward	5'-AGCATGTCAGGATTAGCCGA-3'
BMP4 Reverse	5'-TGGAGATGGCACTCAGTTCA-3'
BMP6 Forward	5'-CAGCCTGCAGGAAGCATGAG-3'
BMP6 Reverse	5'-CAAAGTAAAGAACCGAGATG-3'
BMP7 Forward	5'-TGGAACATGACAAGGAATTCT-3'
BMP7 Reverse	5'-CATCCAGCGTCTCCACCGAGA-3'
BMP9 Forward	5'-AGAACGTGAAGGTGGATTTCC-3'
BMP9 Reverse	5'-CGCACAATGTTGGACGCTG-3'
TGFb1 Forward	5'-CCCAGCATCTGCAAAGCTC-3'
TGFb1 Reverse	5'-GTCAATGTACAGCTGCCGCA-3'
TGFb2 Forward	5'-CCGCATCTCCTGCTAATGTTG-3'
TGFb2 Reverse	5'-AATAGGCGGCATCCAAAGC-3'
Activin A Forward	5'-TGGGCAAGAAGAAGAAGAAA-3'
Activin A Reverse	5'-CACTCACCCTCGCAGTAGTT-3'
LRG1 Forward	5'-GTTGGAGACCTTGCCACCT-3'
LRG1 Reverse	5'-GCTTGTTGCCGTTCAGGA-3'
